# Seasonal Abundance of Psyllid Species on Carrots and Potato Crops in Spain

**DOI:** 10.3390/insects10090287

**Published:** 2019-09-06

**Authors:** Carlos A. Antolínez, Aranzazu Moreno, Irene Ontiveros, Sandra Pla, María Plaza, Susana Sanjuan, José L. Palomo, M. Jennifer Sjölund, Jason C. Sumner-Kalkun, Yvonne M. Arnsdorf, Colin J. Jeffries, David Ouvrard, Alberto Fereres

**Affiliations:** 1 Facultad de Ciencias Exactas, Naturales y Agropecuarias, Grupo de Investigación Agroambiente y Salud-Microbiota Universidad de Santander, 680003 Bucaramanga, Colombia; 2 Instituto de Ciencias Agrarias (ICA, CSIC), Consejo Superior de Investigaciones Científicas (CSIC), C/Serrano 115 Dpdo, 28006 Madrid, Spain; 3 Agricola Villena, Carretera del Puerto, S/N 3400 Villena, Spain; 4 Centro Regional de Diagnostico, Junta de Castilla y León, 37340 Salamanca, Spain; 5 SASA, Roddinglaw Road, Edinburgh EH12 9FJ, UK; 6 Department of Life Sciences, Natural History Museum, Cromwell Road, London SW7 5BD, UK

**Keywords:** *Candidatus* Liberibacter solanacearum, vector-transmission, *Bactericera trigonica*, *Bactericera nigricornis*, psyllid yellows, vector abundance, zebra chip, population dynamics

## Abstract

Psyllids (Hemiptera: Psylloidea) can transmit the phloem restricted bacterium ‘*Candidatus* Liberibacter solanacearum’ (Lso). In Europe, Lso causes severe losses to carrot and represents a threat to the potato industry. A rising concern is Lso transmission from carrot to potato and within potato, and this has driven the need for monitoring populations of psyllid species which could serve as vectors on both crops. This would provide a fundamental understanding of the epidemiology of Lso. Different sampling methods were used to survey populations of psyllid species in commercial carrot and potato fields in central and eastern mainland Spain from 2015 to 2017. Two psyllid species, *Bactericera* *trigonica* and *Bactericera* *nigricornis* were found on carrot and potato crops. In carrot fields the most abundant species was *B.* *trigonica* (occurring from crop emergence to harvest); whereas in potato crops the most abundant psyllid species was *B.* *nigricornis*. Depending on field location, the maximum psyllid populations occurred between June and October. Since *B.* *nigricornis* was found on both carrot and potato and is the only psyllid species able to feed and reproduce on both these crops in Europe, there is the potential risk of Lso transmission from carrot to potato.

## 1. Introduction

Vegetable crop production is a major part of European Union (EU) agriculture, accounting for 13.7% of its agricultural output [1]. Carrot (*Daucus carota* subsp. *sativus* (Hoffm.)) and potato (*Solanum tuberosum* L.) are among the most important vegetable crops in the EU. In 2017, carrot and potato production within the EU was estimated at 5.8 and 62 million tonnes respectively [1]. In recent years psyllids (Hemiptera: superfamily Psylloidea) have emerged as important pests that threaten carrot and potato production in different regions of the world including Europe [2].

In Europe various psyllid species affect carrot production. The predominant species of economic concern are *Trioza apicalis* Foerster, 1848 in northern Europe, and *Bactericera trigonica* Hodkinson, 1981, in the Mediterranean region. Both species cause direct damage to carrot and other Apiaceae and are vectors of the phloem restricted bacterium ‘*Candidatus* Liberibacter solanacearum’ (Lso) [3,4,5]. In carrots, the main disease symptoms of Lso are purple or yellow leaf discoloration and root reduction [5]. The incidence of Lso infected plants depends on the growing zone, vector activity and environmental conditions, but respectively, 90% and 40% of the growing crop have been reported affected in carrot fields in Spain and Tunisia [3,6]. Lso has been mainly associated with Apiaceae crops in Europe, however, naturally Lso-infected potato plants have recently been reported in Spain and Finland [7,8]. It is still uncertain how these potatoes became infected because of the absence of the main vector of potato-associated Lso haplotypes, *Bactericera cockerelli* (Šulc, 1909), the potato psyllid. Where *B. cockerelli* is present, such as in North and Central America and New Zealand [9], Lso affects a wide number of crops in the Solanaceae and it is especially important in potato where it causes a vegetative disorder called “zebra chip” [10,11]. Zebra chip is characterized by a striped pattern of necrosis in tubers, which becomes more evident when chips are fried. As a result, potato chips processed from infected tubers are commercially unacceptable [11] and this has led to the abandonment of entire potato fields and huge economic losses to the potato industry in North and Central America [10,11,12].

*Bactericera cockerelli* is an endemic pest of North and Central America and was introduced to New Zealand and Australia [11,13,14,15]. The geographical distribution of *B. cockerelli* does not overlap with the carrot psyllids *B. trigonica* or *T. apicalis* in Europe [11,16]. Therefore, despite the contiguous cultivation of carrot and potato crops in Europe, current experimental evidence suggests that the risk of transmission of Lso from carrot to potato by *B. trigonica* or *T. apicalis* is very limited. This is because these carrot psyllids are unable to colonize and continuously feed from the phloem of potato plants [8,17]. Despite this low risk of cross transmission, Lso is still of concern to potato producers in Europe because other psyllid species could potentially transmit Lso to, and within, potato especially in Mediterranean countries. For example, *B. nigricornis* Foerster, 1848, which is closely related to *B. trigonica* has been reported on carrot and potato crops [18,19,20,21], and has been tested positive for Lso haplotype E in the field [22]. This potential vector might represent a threat to European carrot and potato production, but very little is known about its population dynamics.

To assess the potential risk of Lso transmission, studies of the population dynamics of vectors and potential vectors of Lso in carrot and potato crops in European countries are urgently required. Furthermore, despite the economic losses associated with carrot psyllids in Mediterranean countries, the population dynamics of these insects are poorly documented. This information is important because management of Lso and its reduction are primarily based on the following: the control of vector populations [11,23]; timing of insecticide application based on psyllid arrival into a crop; and psyllid population peak density [24]. To date, just one study has monitored psyllid populations in mainland Spain, reporting *B. trigonica*, *B. tremblayi* Wagner, 1961, and *B. nigricornis* in celery and only *B. trigonica* in carrots in Tenerife [22]. The same study also reported three limited surveys: (i) in carrots in mainland Spain where these psyllids were found; (ii) in potato in mainland Spain where *B. nigricornis* and *B. trigonica* were found and; (iii) in potato in Tenerife where only *B. trigonica* was found. However, monitoring of psyllid populations during the complete carrot and potato growing season has not been reported in mainland Spain or other Mediterranean countries. Since the information of vector abundance is fundamental to the design of effective management practices to control Lso spread, we monitored the seasonal abundance of psyllids on carrots and potato crops in mainland Spain over three consecutive years using various sampling methods.

## 2. Materials and Methods

### 2.1. Psyllid Sampling Locations

Surveys were conducted over three consecutive years from 2015 to 2017. In the summer of 2015 one single sampling of adult psyllids was performed by sweep net. Sampling was performed in one carrot field and one potato field, both located in Gomezserracín (Segovia, Spain). From May to October of 2016 and 2017 three different carrot fields located in Gomezserracín, Íscar (Valladolid, Spain) and Villena (Alicante, Spain) were sampled. For the potato cultivation cycle (May to September) of 2016 one field located in Aldearrubia (Salamanca, Spain) and another field located in Gomezserracín were sampled. In 2017, only the potato field located in Aldearrubia was available for sampling. For potato and carrot in 2016 and 2017, sampling was conducted every two weeks from crop emergence to harvest using three different sampling methods: sweep net, horizontal green tile water traps and visual inspection. In the potato field located in Aldearrubia adult psyllids were also caught in 2017 using a 12.2 m suction trap. Details of field locations and cultivars grown are shown in Table 1. The size of carrot and potato fields sampled were approximately 1.5 ha. The average temperatures throughout the carrot and potato cultivation cycle for each of the sampling sites were obtained from Agencia Estatal de Meteorologia, AEMET (climatic information system of the Spanish government) [25]. Other environmental factors such as relative humidity and precipitation were analysed, however no significant correlations with insect densities were found.

### 2.2. Sweep Net Sampling

The carrot and potato canopies were sampled for adult psyllids using a telescopic folding sweep net (NHBS Ltd., 1-6 the Stables, Fort Road Totnes, UK). Each sample consisted of ten consecutive sweeps along a surface of 2 m^2^ at ten different points, covering a total distance of 100 m, to obtain a total of ten samples per sampling site and date. Quadrants where psyllids were sampled were not treated with insecticide. Sweep net samples were transferred to plastic ziploc^®^ bags, taken to ICA-CSIC in Madrid-Spain, and frozen at −20 °C until taxonomic identification.

### 2.3. Horizontal Green Tile Water Traps

Horizontal green water tile traps (also called Irwin traps) [26] were selected for this study because they are neutral traps. They are similar to the canopy background color and were designed to specifically collect alate insects (mainly aphids) that land on a row crop such as soybeans [26,27]. Traps used in this study consisted of a methacrylate container (16.5 × 16.5 × 4.5 cm) and a square ceramic tile (15.5 × 15.5 cm) (Cambridge 815 from Cambridge tile C., PO Box 15071, Cincinnati, OH 46215, USA), which was placed inside a container. The green ceramic tile has an absorbance spectrum similar to the soybean canopy but has been used to monitor sap sucking insects landing on several other row crops such as pepper, lettuce and broccoli [28,29,30,31]. The container was filled with a 50% solution of ethylene glycol in water. A second container was placed below the tile in order to avoid losing insect samples in case of heavy rain. The trap was always placed at canopy level. Insects captured were collected every two weeks and the solution in the container was changed at the same time. The trap content was filtered using a funnel and the collected insects were preserved in 70% ethanol until taxonomic identification. Only insects from the Psylloidea were identified to species level. One trap per sampling site was used in all of the carrot and potato fields surveyed. Traps were located approximately 20 m from the edge of the crop.

### 2.4. Visual Inspection

In the carrot fields, twenty whole plants were visually inspected for eggs and immatures at each sampling site at two-week intervals, from seedlings to harvest. Plants were randomly selected by walking in a zig-zag pattern in a diagonal across the field covering a distance of 100 m. A 0 to 4 scale was used to rate immature and egg density: 0 = 0; 1 = 1-4; 2 = 5–20; 3 = 21–50; 4 = more than 50. Infestation of psyllids on potato fields was not scored because immatures were not detected on plants.

### 2.5. Suction Trap

The daily psyllid flight activity was monitored using a 12.2 m high suction trap. The suction trap was located at Aldearrubia in the same potato field selected for sampling psyllids by other methods. The suction trap operated during the summer months of 2017 (15 June 2017–31 August 2017). The trap contents were removed every 24 h at the same time daily and the insects preserved in 70% ethanol until taxonomic identification.

### 2.6. Psyllid Identification

Adult psyllids were identified to species level based on morphological characteristics following the taxonomic keys of Burckhardt and Freuler (2000) and Hodkinson (1981) [18,32]. Suction trap samples and specimens collected in the field not identified morphologically were identified by DNA barcoding at SASA, Edinburgh, United Kingdom. Psyllid DNA was extracted using a non-destructive method [33] in which the insect was pierced through the thorax and abdomen using a 0.14 mm steel pin, and the DNA extracted and purified using the DNeasy Blood & Tissue Kit (QIAGEN, Venlo, The Netherlands) following the manufacturer’s instructions. New pins were used for each psyllid specimen. Psyllid exoskeletons were preserved in 95% ethanol and 5% glycerol and then stored in plastic vials as voucher specimens. Two regions of psyllid DNA were amplified: Cytochrome oxidase I (COI) region using primers LCO1490 and HCO2198 according to [34] and the internal transcribed region 2 (ITS2) using primers CAS5p8sFcm and CAS28sB1d according to [35]. Successful amplifications of gene regions were verified by electrophoresis on a 1% agarose gel and visualized using an UV illuminator. PCR products were then cleaned using ExoSAP-IT™ (Affymetrix USB Products) treatment and ethanol precipitation. For the sequencing reaction “BigDye™ Terminator v3.1 Cycle Sequencing Kit” (NimaGen BV, Nijmegen, The Netherlands) was used and products were sequenced via Sanger capillary sequencing on a “3500 Genetic Analyser” (Applied Biosystems, Foster City, CA, USA). Both DNA strands of the target gene regions were sequenced using forward and reverse primers separately. Each contig was aligned to create a consensus sequence using a CLUSTAL-W algorithm on “Geneious 10” software. Sequences were then identified using BLAST (basic local alignment search tool) against GenBank, BOLD (barcode of life data systems) and SASA’s psyllid DNA barcoding database to determine the closest match. Sequences with 98%–100% identity scores to species in the SASA psyllid database were deemed to be the same species. The corresponding sequences were uploaded to GenBank (see Table S1 and Table 5 for GenBank accession numbers).

## 3. Results

### 3.1. Sweep Net Sampling

*Bactericera trigonica* was the most abundant species found in all the carrot fields surveyed across years and locations, followed by *B. nigricornis* (Table 2 and Table 3). In contrast, *B. nigricornis* was the most abundant species in the potato fields surveyed from 2015 to 2017 (Table 2 and Table 3), except for one potato field surveyed in 2016 located in Gomezserraín where *B. trigonica* was the most abundant species (Table 3). For carrot and potato, other psyllid species were occasionally observed and although not included in the analysis, 2016 data are provided in Table S2.

**Figure 1:**
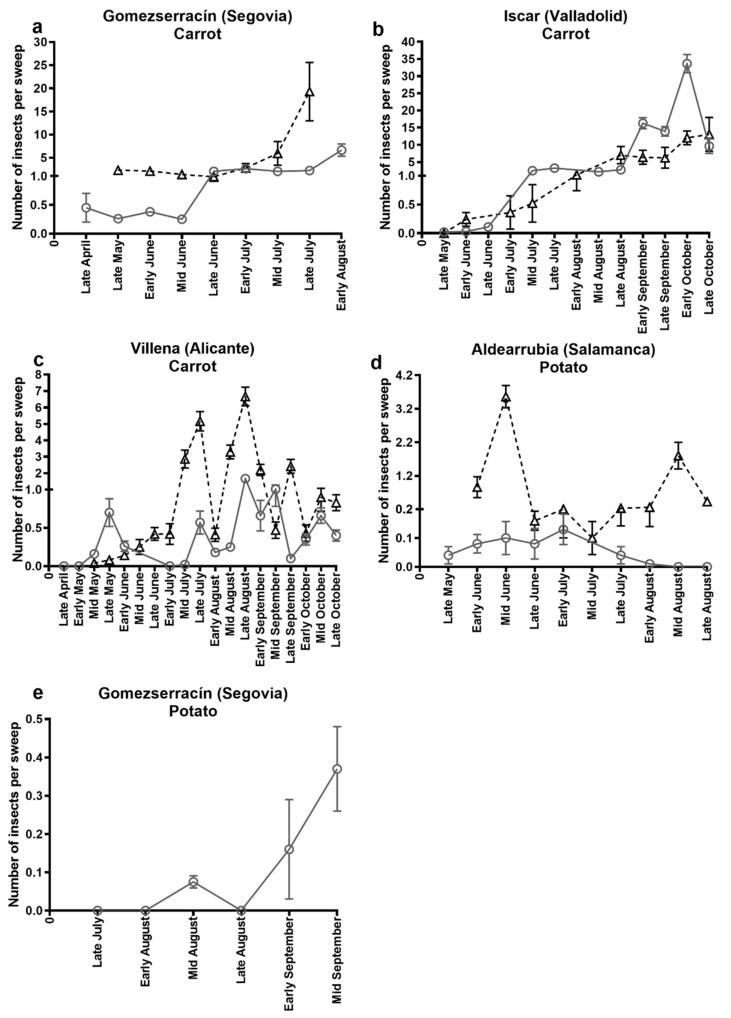
Mean number of psyllids (*B. trigonica* + *B. nigricornis*) collected per sweep in 2016 (grey circles and solid line) and 2017 (black triangles and dashed line). Samples were collected in carrot fields, (**a**) Gomezserracín, (**b**) Íscar, (**c**) Villena or in potato fields, (**d**) Aldearrubia and (**e**) Gomezserracín. *Y*-axis values represent the mean number of insects per sweep and *X*-axis represents collecting dates.

In the carrot fields surveyed, psyllid populations followed similar trends between 2016 and 2017 (Figure 1). Psyllid population peaks varied depending on where the carrots were grown. In the carrot field located in Gomezserracín the first psyllids appeared in late April (2016) and late May (2017) and were at low population levels until late June (2016) and early July (2017) when they showed a gradual increase, reaching a maximum population peak in early August 2016 (mean number of psyllids per sweep 6.6 ± 1.32) and late July 2017 (mean number of psyllids per sweep 19.3 ± 6.30) (Figure 1a). In the carrot field located in Íscar, the first psyllids appeared in late May (2016 and 2017) and then increased gradually to a maximum population peak in October (early October 2016, mean number of psyllids per sweep 33.6 ± 2.67 and late October 2017, mean number of psyllids per sweep 13.0 ± 4.91) (Figure 1b). For the carrot field located in Villena, the first psyllids arrived at the crop in mid-May and then population density varied throughout the cultivation cycle reaching a maximum population peak in late August (late August 2016, mean number of psyllids per sweep 1.7 ± 0.24 and late August 2017, mean number of psyllids per sweep 6.7 ± 0.56) (Figure 1c).

In the two potato fields surveyed, maximum population peaks also varied depending on the field location. In the field in Aldearrubia, the first psyllids were detected in late May 2016 and early June 2017 (Figure 1d) with maximum population peaks in early July 2016 (mean number of psyllids per sweep 0.1 ± 0.05) and in mid-June of 2017 (mean number of psyllids per sweep 3.56 ± 0.33) (Figure 1d). In the potato field located in Gomezserracín the first psyllids were not observed until late July with a maximum population peak in September, when the crop was harvested (mean number of psyllids per sweep 0.4 ± 0.11) (Figure 1e).

### 3.2. Horizontal Green Tile Water Trap Sampling

**Figure 2:**
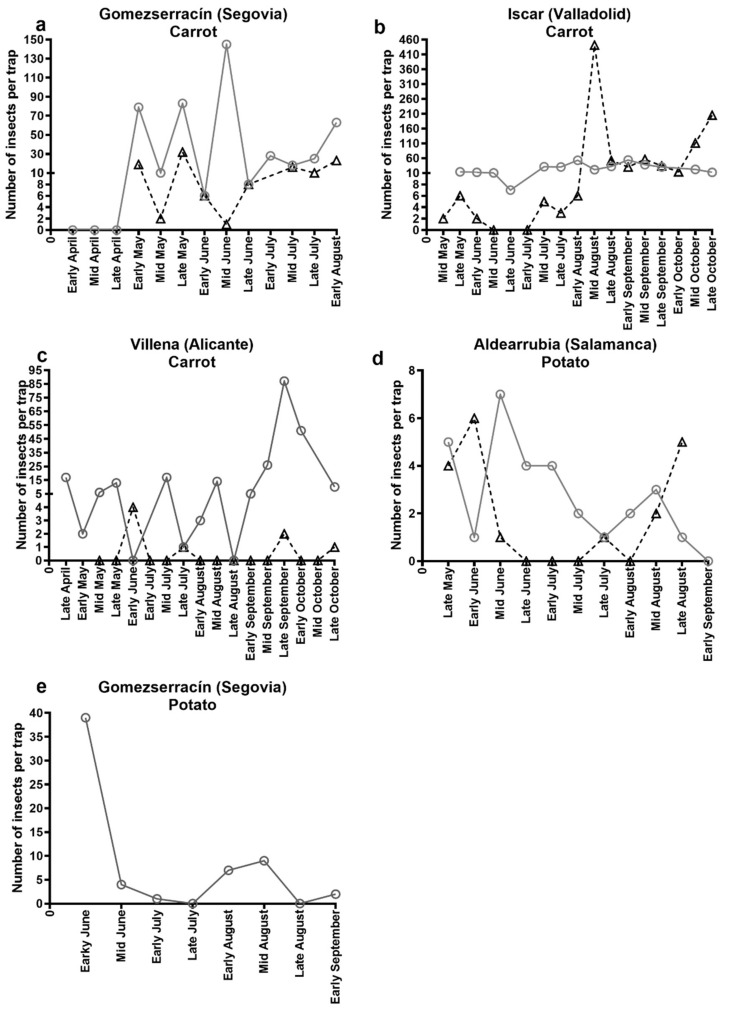
Total number of psyllids (*B. trigonica* + *B. nigricornis*) collected by horizontal green water tile traps in 2016 (grey circles and solid line) and 2017 (black, triangles and dashed line). Samples were collected in carrot fields, (**a**) Gomezserracín, (**b**) Íscar, (**c**,**d**) Villena or in potato fields, (**e**) Aldearrubia and (**f**) Gomezserracín.

Green tile traps showed *B. trigonica* to be the most abundant psyllid species in all carrot fields sampled (Table 4). *Bactericera nigricornis* were also recorded in traps located in all carrot fields, however they were found at very low numbers (Table 4). In Gomezserracín, the first psyllid adults were found in early May and adults were observed until the last sampling date in early August in both 2016 and 2017 (Figure 2a). The maximum population peaks were observed in mid-June 2016 (total number of psyllids per trap = 145) and late May 2017 (total number of psyllids per trap = 32). In Íscar, adult psyllids were observed from the first samplings in May 2016 and 2017 (Figure 2b) with maximum population peaks in early August 2016 (total number of psyllids per trap = 54) and in mid-August 2017 (total number of psyllids per trap = 441). In Villena, psyllids were present in late April 2016 with a maximum population peak in early September (total number of psyllids per trap = 87) (Figure 2c). This contrasted with 2017, when psyllids were absent at the first sampling dates in mid-May and were not caught until early June (total number of psyllids per trap = 5). Overall the numbers of psyllids collected in Villena for the carrot cultivation cycle of 2017 were very low.

For all potato fields, the green tile traps showed *B. nigricornis* to be the most abundant species observed in 2016 and 2017 (Table 4). *Bactericera trigonica* was also present but in low numbers and not observed in all surveys. It was observed in Gomezserracín in 2016, as well as Aldearrubia in 2017 but not 2016 (Table 4). In the potato field located in Aldearrubia, psyllids were observed from May to August, showing the maximum peak in mid-June 2016 (total number of psyllids per trap = 7) and in early June 2017 (total number of psyllids per trap = 6) (Figure 2d). In Gomezserracín, psyllids were observed from the first sampling date in early June to early September. The maximum population peak occurred in early June (total number of psyllids per trap = 39), and then the population decreased (Figure 2e). The list of other psyllid species (*B. tremblayi*, *Trioza remota* Foerster, 1848, and *Trioza urticae* Linné, 1758) occasionally caught in potato is provided in Table S2.

### 3.3. Visual Inspection

**Figure 3:**
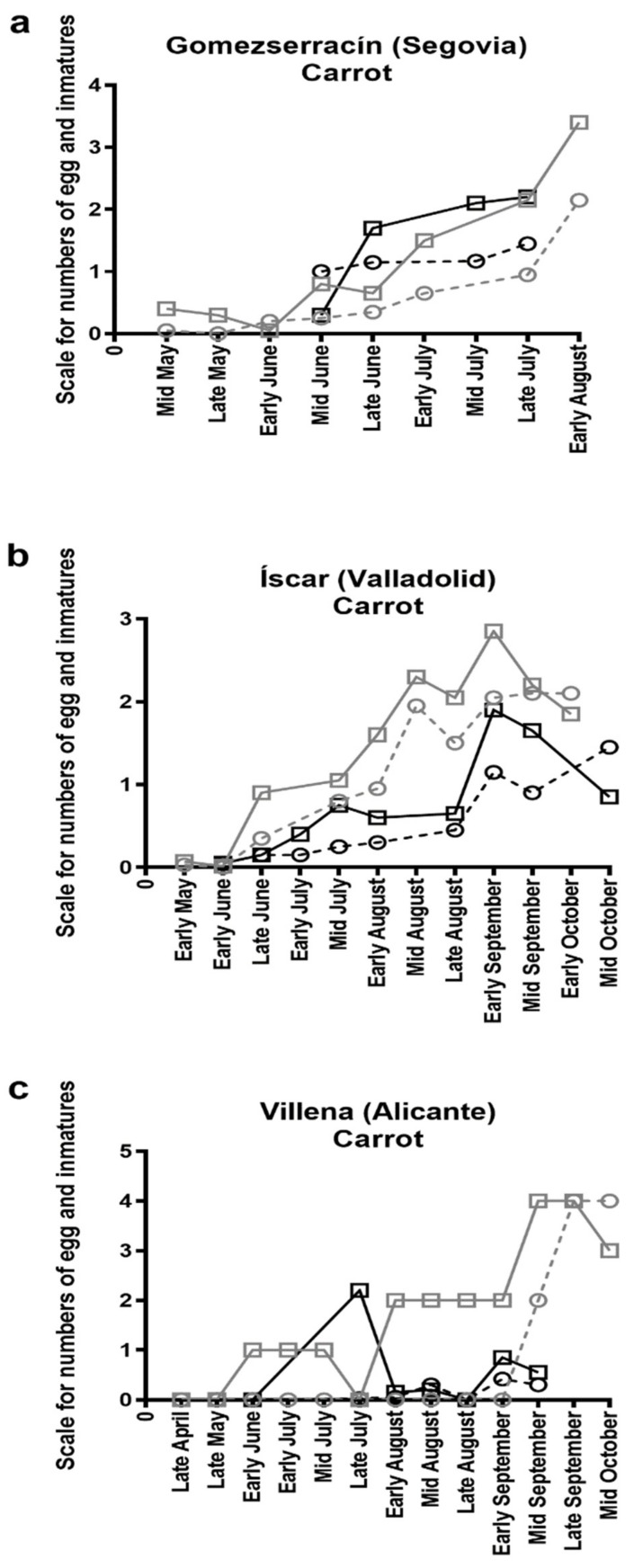
Number of eggs (squares and solid line) and immatures (circles and dashed line) found in 2016 (grey) and 2017 (black) in commercial carrot fields in (**a**) Gomezserracín, (**b**) Íscar and (**c**) Villena. *Y*-axis represents scale values. Scale for eggs and nymphs used was: 0 = 0, 1 = 1–4, 2 = 5–20, 3 = 21–50, 4 = more than 50.

The number of eggs and immatures sampled in carrot fields are shown in Figure 3. Overall numbers of eggs and immatures increased gradually and reached their highest levels some days earlier than the maximum population peak observed for adults: Early August in Gomezserracín, early September in Íscar and mid to late September in Villena. In potato, due to the very low numbers of psyllids present in both fields, visual inspection was not performed. Despite obtaining visual identification to species level using adult psyllids, this was not possible by using eggs. Therefore, it is not known if the eggs found belong to both psyllid species identified in the adult sampling or only to one of them.

### 3.4. Suction Trap

The most abundant psyllid species in the suction trap in Aldearrubia in 2017 were *Blastopsylla occidentalis* Taylor, 1985, followed by *Ctenarytaina spatulata* Taylor, 1997. Just three individuals of *B. trigonica* were caught and *B. nigricornis* was not found (Table 5). According to these data the psyllid species present at crop level differed to those captured at 12.2 m high and may have been migrating species not capable of colonising potato.

## 4. Discussion

Previous studies have shown that only the psyllid species that are able to colonize a given crop efficiently transmit Lso [8,17,36]. Therefore, knowledge of psyllid populations in European carrot and potato crops is important to understand Lso spread in the field and to assess the potential risk of Lso introduction to potato crops.

Using different sampling techniques (sweep net, tile water traps and visual inspection) we observed that *B. nigricornis* and *B. trigonica* adults were present on potato and carrot crops, with *B. nigricornis* and *B. trigonica* most abundant on potato and carrot respectively. These results are consistent with previous work where *B. trigonica* was found to be the predominant species in celery and carrot in mainland Spain and Tenerife [22]. However, Teresani et al. [22], using sticky traps, did not report *B. nigricornis* on carrots except for a field in La Rioja (38 psyllids from one sampling in 2012) and with very low populations in celery (2 psyllids in total from 3 samplings) and potato (2 psyllids from one sampling). Our results are also consistent with field surveys in carrot crops conducted in Tunisia were *B. nigricornis* and *B. trigonica* were the only two psyllid species found [21]. On the other hand, psyllid species surveyed by the 12.2 m suction trap on potato differed from those obtained by the other sampling methods used. Psyllids caught by the suction trap were probably migrating species that were not associated with carrot or potato crops in this study. This is consistent with recent work from Germany where no carrot psyllids were caught by using a similar suction trap in a carrot field [37]. This suggests that sampling methods performed at the level of the crop canopy gives a more accurate estimate of the species present on carrot and potato crops than suction traps.

The psyllid population peaks observed in carrot using sweep nets occurred from June to October and although the timing of these peaks varied according to location it was consistent at each location in 2016 and 2017. These population peaks were correlated with mean temperatures recorded for each of the locations sampled during 2016 and 2017 (Figures S1–S3). The arrival of the first psyllid adults at each location occurred when temperatures were above 15 °C. Psyllid maximum population peaks were observed when mean temperatures were close to 25 °C (Figures S1 and S2). Despite all quadrants used in our study not being treated with insecticides, sprays applied to the surrounding crops could have affected our results. However, similar population peaks observed for each sampling site in different years suggests that treatments performed near our sampling sites had little effect on the results obtained. Our data slightly differs from that reported by Teresani et al. [22], who observed psyllid population peaks from April to August on celery crops in Villena and in July for carrot crops in Tenerife (Canary Islands). The most abundant species found in our work, differed from the species *B. nigricornis*, *Trioza anthrisci* Burckhardt, 1986, and *T. apicalis,* collected from carrot fields in Valais, Switzerland [18]. In their survey, *B*. *nigricornis* was the most abundant species in two of the three carrot fields sampled; whereas *B. trigonica* was found in very low numbers. The very different environmental conditions between the two regions, Spain and Switzerland, might explain differences in the psyllid populations. The current distribution of *B. trigonica* in the Mediterranean region suggests that the climatic conditions of this zone might be more favorable for this species than other species such as *B. nigricornis*, *T. anthrisci* or *T. apicalis*. To date, the latter two species have not been reported in Spain and are commonly associated with Apiaceae plants in cooler and more humid regions in Northern and Central Europe [38,39].

Adults of *B. trigonica* were found at the first samplings in carrot fields. However, it is not known if these were adults that migrated from other zones or were already present nearby. For this species, migration behavior has not been studied and it is uncertain if adults overwinter on plant species different to those commonly reported as hosts. In Central Europe, Burckhardt and Freuler [18] have suggested that *B. trigonica* can overwinter as adults on conifers or evergreen shrubs. However, in the regions where these surveys were conducted *B. trigonica* may also migrate from carrots or celery crops that are widely present, because of the favorable climatic conditions for year-round crop production. In contrast to *B. trigonica*, *B. nigricornis* was absent from the first samplings suggesting that migration from other hosts or fields occurs later than *B. trigonica*. Although *B. nigricornis* is present in many countries [40] little is known about its biology and dispersal ability it has been mentioned as a conifer overwintering species [41]. Furthermore, *B. nigricornis* has been reported as a polyphagous species able to feed on wild species belonging to the Amarantaceae, Boraginaceae, Brassicaceae, Liliaceae, Papaveraceae and Solanaceae [32]. Thus, further research on the life cycle of this species is needed to identify its overwintering hosts or migration habits from wild host species under Mediterranean conditions.

Although eggs and immatures of *B. nigricornis* were not observed during visual inspections, previous reports have confirmed that this psyllid species is able to reproduce on potato [32] and has been reported causing severe yield losses in Iran [20]. Interestingly, *B. nigricornis* has not caused economic damage to potato crops in Spain, perhaps due to its low population density on potato. Nevertheless, monitoring its populations is highly recommended as a preventive measure to avoid potential outbreaks.

The high numbers of *B. trigonica* detected in the potato field located in Gomezserracín in 2016 were unexpected, as previous studies have suggested that this species is only restricted to the Apiaceae and does not breed on potato [17,19]. However, since no immature stages were observed and the arrival dates of *B. trigonica* to potatocoincided with the harvesting of carrots in the same region, we suggest the active movement of *B. trigonica* into potato occurred when carrots were no longer available. Despite not being able to colonize potato adults of *B. trigonica* might be able to survive on “food plants” [42] for a short time as has been reported for related psyllid species such as *B. cockerelli* [36].

Transmission of vector-borne pathogens is affected by several factors with vector activity (the abundance of insects landing on the crop) and vector propensity being especially important [27]. High populations of *B. trigonica* on carrots reported here and evidence of *B. trigonica* being an efficient vector of Lso [17,43,44], could explain the high Lso incidence reported in carrot fields in Spain [3,6]. However, for potato, the low number of *B. trigonica* found on the crop and previous experimental evidence suggests that the risk of Lso transmission from carrot to potato by this psyllid species is negligible [17].

On the other hand, since *B. nigricornis* was consistently found on both crops and to date is the only known European psyllid able to reproduce on potato, there is a potential risk of Lso transmission from carrot to potato. Although the vector efficiency of *B. nigricornis* has not been assessed, previous field work has shown that it can become naturally infected with Lso haplotype E [22]. Preliminary work being conducted at ICA-CSIC suggests that infected *B. nigricornis* is able to transmit Lso haplotype E to carrot and potato and cause typical symptoms of disease (Ontiveros et al., in preparation). However, even if Lso transmission is possible by *B. nigricornis*, the frequency and significance of this transmission remains uncertain under field conditions since the observed population numbers for this species were low. Thus, further research aimed at understanding Lso primary transmission from carrot to potato and secondary transmission from potato to potato plants by *B. nigricornis* under field conditions in Europe is needed.

## 5. Conclusions

The psyllid *B. trigonica* was the most abundant species caught on carrot whereas *B. nigricornis* was the most abundant on potato. Overall, our research in Spain contributes significantly to understanding population dynamics of psyllids in carrot and potato crops which is required for Lso pest risk analysis by National and Regional Plant Protection Organizations.

## Figures and Tables

**Table 1:** Location and information of carrot and potato fields surveyed by different sampling methods during the cultivation cycles of 2015, 2016 and 2017 in Spain.

Crop	Cultivar	Field Location	Province	Latitude	Longitude	Altitude
Carrot	Bangor	Gomezserracín	Segovia	41°17′24″ N	4°19′32″ W	804
	Bangor	Íscar	Valladolid	41°20′05″ N	4°32′13″ W	750
	Soprano	Villena	Alicante	38°38′47″ N	0°55′47″ W	450
Potato	Monalisa	Aldearrubia	Salamanca	49°59′11.2″ N	5°29′10.1″ W	812
	Monalisa	Gomezserracín	Segovia	41°17’20″ N	4°19‘03″ W	805

**Table 2:** Survey of adult psyllids associated with carrot and potato fields in Gomezserracín (Segovia) Spain conducted in July 2015.

Crop	Location	Sampling Method	Psyllid Species	Date of Sampling	Total Number of Insects
Carrot	Gomezserracín	Sweep net	*B. trigonica*	02/07/2015	20
			*B. nigricornis*	02/07/2015	1
Potato	Gomezserracín	Sweep net	*B. nigricornis*	02/07/2015	5
			*B. tremblayi*	02/07/2015	3

**Table 3:** Percentage of psyllid species found by sweep net sampling in carrot and potato fields surveyed in 2016 and 2017.

Crop	Location	Year					
		2016			2017		
		% *B. trigonica*	% *B. nigricornis*	Total number of psyllids	% *B. trigonica*	% *B. nigricornis*	Total number of psyllids
Carrot	Gomezserracín	97	3	1763	93	7	3069
	Íscar	98	2	8349	98	2	4683
	Villena	94	6	654	92	8	4750
Potato	Aldearrubia	2	98	52	11	89	249
	Gomezserracín	82	18	73			

**Table 4:** Percentage of psyllid species in horizontal green tile water traps in carrot and potato fields surveyed in 2016 and 2017.

Crop	Location	Year					
		2016			2017		
		% *B. trigonica*	% *B. nigricornis*	Total number of psyllids	% *B. trigonica*	% *B. nigricornis*	Total number of psyllids
Carrot	Gomezserracín	97	3	534	74	26	121
	Íscar	94	6	365	99	1	1027
	Villena	99	1	266	75	15	8
Potato	Aldearrubia	0	100	30	10	90	21
	Gomezserracín	29	61	64			

**Table 5:** Psyllid species collected by the suction trap in the potato field in Aldearrubia Salamanca. CO1 gene regions were successfully sequenced from all psyllid specimens. Internal transcribed region 2 (ITS2) sequencing was successful for all except the *C. melanoneura* and *Ct. spatulata* specimens.

Genus	Species	Authorship	Quantity	Accession # ITS	Accession # CO1	Date Collected
*Arytainilla*	*gredi*	(Ramirez Gomez, 1956)	1	MN316677	MN272123	16/06/2017
*Bactericera*	*trigonica*	Hodkinson, 1981	1	MN316680	MN272129	16/06/2017
			1	MN316691	MN272143	18/08/2017
			1	MN316694	MN272148	30/08/2017
*Blastopsylla*	*occidentalis*	Taylor, 1985	1	-	MN272126	16/06/2017
			1	MN316682	MN272132	22/06/2017
			1	MN316683	MN272134	05/07/2017
			4	MN316684-MN316687	MN272135-MN272138	10/07/2017
			1	MN316688	MN272142	17/08/2017
			1	MN316690	MN272144	23/08/2017
			3	MN316691-MN316693	MN272145-MN272147	25/08/2017
*Cacopsylla*	*melanoneura*	(Foerster, 1848)	2	-	MN272124-MN272125	16/06/2017
			1	-	MN272131	17/06/2017
*Ctenarytaina*	*spatulata*	Taylor, 1997	1	-	MN272133	24/06/2017
			1	-	MN272139	10/07/2017
			1	-	MN272140	12/07/2017
			1	-	MN272141	16/07/2017
*Livia*	*crefeldensis*	Mink, 1855	1	MN316678	MN272127	16/06/2017
*Trioza*	*galii*	Foerster, 1848	1	MN316679	MN272128	16/06/2017
			1	MN316681	MN272130	17/06/2017

## Supplementary Materials

The following are available online at https://www.mdpi.com/2075-4450/10/9/287/s1, Table S1: List of psyllid species occasionally found in the carrot and potato fields surveyed in 2016. Table S2: List of psyllid species occasionally found in the carrot and potato fields surveyed in 2016. Figure S1: Mean number of psyllids (*B. trigonica* + *B. nigricornis*) collected per sweep net in 2016 (gray bars) or 2017 (black bars). Samples were collected in carrot fields, (a) Gomezserracín, (b) Íscar, (c) Villena or in potato fields, (d) Aldearrubia and (e) Gomezserracín. Blue lines represent average temperature in 2016 and orange lines represent average temperature in 2017. Figure S2: Total number of psyllids (*B. trigonica* + *B. nigricornis*) collected by green tile traps in 2016 (gray bars) or 2017 (black bars). Samples were collected in carrot fields, (a) Gomezserracín, (b) Íscar, (c) Villena or in potato fields, (d) Aldearrubia and (e) Gomezserracín. Blue lines represent average temperature in 2016 and orange lines represent average temperature in 2017. Figure S3: Number of eggs and immatures found in 2016 and 2017 in commercial carrot fields in (a) Gomezserracín, (b) Íscar and (c) Villena. The *Y*-axis right represents scale values. Scale for eggs and nymphs used was as follows: 0 = 0, 1 = 1–4, 2 = 5–20, 3 = 21–50, 4 = more than 50. *Y*-axis left represents average temperatures.
